# The Royal Victoria Infirmary, Newcastle

**Published:** 1913-02-15

**Authors:** 


### THE ROYAL VICTORIA INFIRMARY, NEWCASTLE.

### A Little Known Literary Association.

As has previously been noted in these columns, among the fashions which have passed is that of inscribing the walls of hospitals with the names of benefactors and the exact amounts of their donations. The entrance hall of any old-established county infirmary—the town ones have generally had to be pulled down and rebuilt on another site, or altogether remodelled—is very likely to show a blue ground covered with gold letters and figures, with, it may be, a large oil painting of the gentleman who heads the list. Nowadays our taste seems, to borrow one of Mr. Henry James' invaluable adjectives, less florid. Not, probably, that we are really more modest, for in present-day newspapers and hospital literature charity gets published much more widely than it could be by means of mural tablets. The reason for the blank spaces customarily seen now may be that the quick growth of hospitals, following on the advance in surgery due to Listerism, made it very clear to each house committee that if everyone who gave fifty pounds (and some of these old recorded donations were no more) had to have his name painted up, the interior of the building would ere long become one expanse of lettering. The custom, of course, is one of those things which, once useful, the subsequent trend of events has rendered superfluous. But there may remain to such inscriptions many kinds of extraneous interest—antiquarian, historical, legal, biographical, literary, and so forth. A literary association, although of a rather unimportant kind, but hitherto unsuspected, or at least not described, we thus find in an institution lately reported on in The Hospital, one of the finest of provincial hospitals—namely, the Royal Victoria Infirmary at Newcastle-upon-Tyne. There is affixed to the rebuilt walls of its out-patient department a large wooden tablet which sets forth in gold letters an account of a certain Pigg Charity. Some lands and houses in the neighbourhood, it appears, were left by one John Pigg, in the seventeenth century, to be devoted, subject to one or two small deductions, to the benefit of the sick poor of Northumberland and Durham; and in 1832 the infirmary was given the disposal of these funds.

An ordinary phenomenon enough, of course; how many of our readers, seeing this tablet, would be reminded of the following quotation in the true Tyneside dialect, and how many will recognise it?

> "Ar de believe, though, gin ar had me dues, ar'd be a gen'lman this day—only ye see, sir, you see, ma fore-elder John, ye see John Pigg, willed away arle wor brass to the Formory, ye see, and left me wi' fairly nout. Gin ye gan to the Newcassel Formory, ye'll see arle aboot it, in great goud letters, clagged agin the walls."

The book from which the above is taken is *Handley Cross*, not an immortal work perhaps, but one which, aided by Leech's illustrations, has earned its author Surtees as much as a column in the *Dictionary of National Biography*. Though of trifling value compared with Izaak Walton's blend of sport and literature, it may be said to rank high amongst what Charles Kingsley called the offspring of a tenth muse. All sportsmen know the tea-dealing master of foxhounds Jorrocks, and his North-Country henchman, doughty James Pigg, whose *ipsissima verba* it is that are quoted. But of the thousands to whom they are familiar very few would expect to find actual corroboration for them. The matter seems worth looking into, and we find from the Northumberland County History Committee's report and other sources that John Pigg was an eccentric Newcastle character, sometime town surveyor, who died in 1688. There is no mention of any disappointed descendants however. His niece, one Ann Rea, was provided for, and ultimately came off with a farm for her portion. And John Pigg was quite unlike his fictitious namesake in character, being noted for his unpopularity, the fervour of his religious opinions, and his aversion to riding on horseback. He provided in his lifetime an additional memorial of himself in the shape of a curious stone pillar on the Great North Road near to Newcastle. It was covered with scraps of Bible texts, and went by the name of Pigg's Folly, being demolished in 1829. An explanation of why Surtees, who was born in Newcastle, and as his books show, possessed intimate knowledge of Northumbrian ways, should have fathered his literary offspring upon this oddity, half Puritan, half figure-of-fun, may be afforded by the more detailed history of the bequest. John Pigg did not leave his property direct to the "Formory," but to certain trustees for the benefit of the local sick poor. But as these trustees died off, no new ones were appointed: the estate came finally into the management of the last survivor, who, it is regrettable to learn, applied the same to his own private use. It was enjoyed by him or his until the year 1832, when the charity was legally re-settled, and the annual residue contributed to the infirmary funds.

Surtees, who by 1838 had succeeded to an estate in Durham only twelve miles from Newcastle (he must not be confused with the Durham historian of the same name), and who from his early experience as a solicitor would be mildly interested in legal transactions, may therefore well have been holding up his neighbour's dishonesty to local derision, while not making the hint so broad as to let outsiders into the secret. In his small sphere, too, he was evidently something of a satirist. At any rate, this curious connection of a detail in what Mr. Kipling enthusiastically calls the story of

> ". . . Jorrocks and his deathless train,
> Pigg, Binjimin, and Arter Xerxes,"

with a matter of fact item of charity accounts, is worth noting by those interested either in the book or the institution. Dr. G. H. Hume's well written and otherwise comprehensive "History of the Newcastle Infirmary" does not mention it.

[Other institutions possessing analogous, but not necessarily literary, associations, are invited to send them to The Hospital for publication.—Ed.]

